# The genome sequence of the March moth, *Alsophila aescularia *(Denis & SchiffermÃ¼ller)

**DOI:** 10.12688/wellcomeopenres.20650.1

**Published:** 2024-02-19

**Authors:** Douglas Boyes, Peter W. H. Holland, University of Oxford and Wytham Woods Genome Acquisition Lab, Darwin Tree of Life Barcoding collective, Wellcome Sanger Institute Tree of Life Management, Samples and Laboratory team, Wellcome Sanger Institute Scientific Operations: Sequencing Operations, Wellcome Sanger Institute Tree of Life Core Informatics team, Tree of Life Core Informatics collective, Darwin Tree of Life Consortium

**Affiliations:** 1 UK Centre for Ecology & Hydrology, Wallingford, England, UK; 2 University of Oxford, Oxford, England, UK

**Keywords:** Alsophila aescularia, March moth, genome sequence, chromosomal, Lepidoptera

## Abstract

We present a genome assembly from an individual male *Alsophila aescularia* (the March moth; Arthropoda; Insecta; Lepidoptera; Geometridae). The genome sequence is 901.6 megabases in span. Most of the assembly is scaffolded into 14 chromosomal pseudomolecules, including the Z sex chromosome. The mitochondrial genome has also been assembled and is 16.67 kilobases in length. Gene annotation of this assembly on Ensembl identified 13,618 protein coding genes.

## Species taxonomy

Eukaryota; Metazoa; Eumetazoa; Bilateria; Protostomia; Ecdysozoa; Panarthropoda; Arthropoda; Mandibulata; Pancrustacea; Hexapoda; Insecta; Dicondylia; Pterygota; Neoptera; Endopterygota; Amphiesmenoptera; Lepidoptera; Glossata; Neolepidoptera; Heteroneura; Ditrysia; Obtectomera; Geometroidea; Geometridae; Alsophilinae; *Alsophila*; *Alsophila aescularia* (Denis & Schiffermüller) (NCBI:txid104486).

## Background

Female flightlessness, with associated loss or reduction of wings, has evolved several times independently in Lepidoptera ( Wahlberg *et al.*, 2010). The March moth *Alsophila aescularia* is a striking example, in which the highly active males have elongate wings and the flightless females completely lack external wings. At rest, the wings of the male are held overlapping and curled around the body; this is a very unusual resting position for moths in the family Geometridae although a similar posture is seen in some other lepidopteran families. The forewings of male *A. aescularia* have a grey-brown ground colour with a distinctive pale scalloped band towards the distal wing margin ( Lawrence Sterne Trust, 2014; NBN Atlas Partnership, 2023).

*A. aescularia* is found across most of Europe, ranging from Scotland and Scandinavia to southern Italy, although there are relatively few records from the Iberian peninsula ( GBIF Secretariat, 2023). There are also scattered records from Ukraine and Russia with an eastern limit at the Ural mountains ( GBIF Secretariat, 2023). As reflected in the common name, the adult moth is active in spring; the majority of records from Britain and Ireland are from February to April, strongly peaking in March ( NBN Atlas Partnership, 2023). Eggs of *A. aescularia* are laid in spring and the larvae feed through late spring and summer before over-wintering at the pupal stage. The larvae are polyphagous, feeding on leaves of deciduous trees including oak ( *Quercus* sp.), sycamore ( *Acer pseudoplatanus*), mountain ash ( *Sorcus aucuparia*) and fruit trees. Occasionally, the species can become a pest in forestry or horticulture, for example in apple orchards in Bulgaria ( Velcheva, 2009).

Here we report a complete genome sequence for the March moth *Alsophila aescularia* determined as part of the Darwin Tree of Life project. The genome sequence of *A. aescularia* will facilitate research into the evolution of sexual dimorphism in wing development and into adaptations to polyphagy, and will contribute to the growing set of resources for studying molecular evolution in the Lepidoptera.

## Genome sequence report

**Figure 1.**
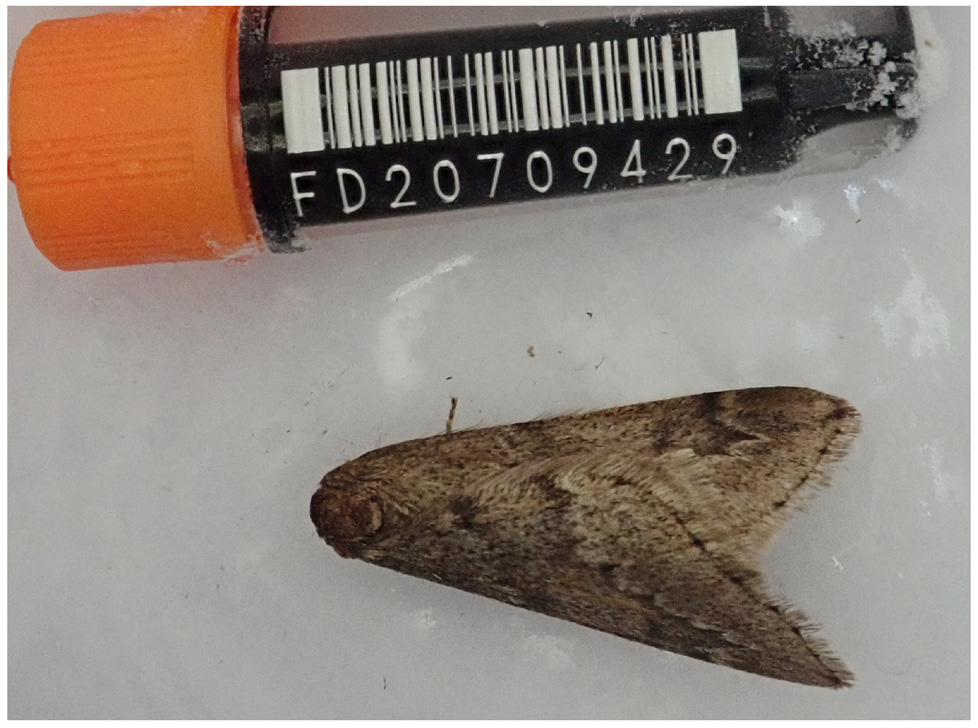
Photograph of the *Alsophila aescularia* (ilAlsAesc1) specimen used for genome sequencing.

The genome was sequenced from one male *Alsophila aescularia* ( Figure 1) collected from Wytham Woods, Oxfordshire, UK (51.77, –1.34). A total of 23-fold coverage in Pacific Biosciences single-molecule HiFi long reads was generated. Primary assembly contigs were scaffolded with chromosome conformation Hi-C data. Manual assembly curation corrected 12 missing joins or mis-joins and removed 2 haplotypic duplications, reducing the scaffold number by 12.5%.

**Figure 2.**
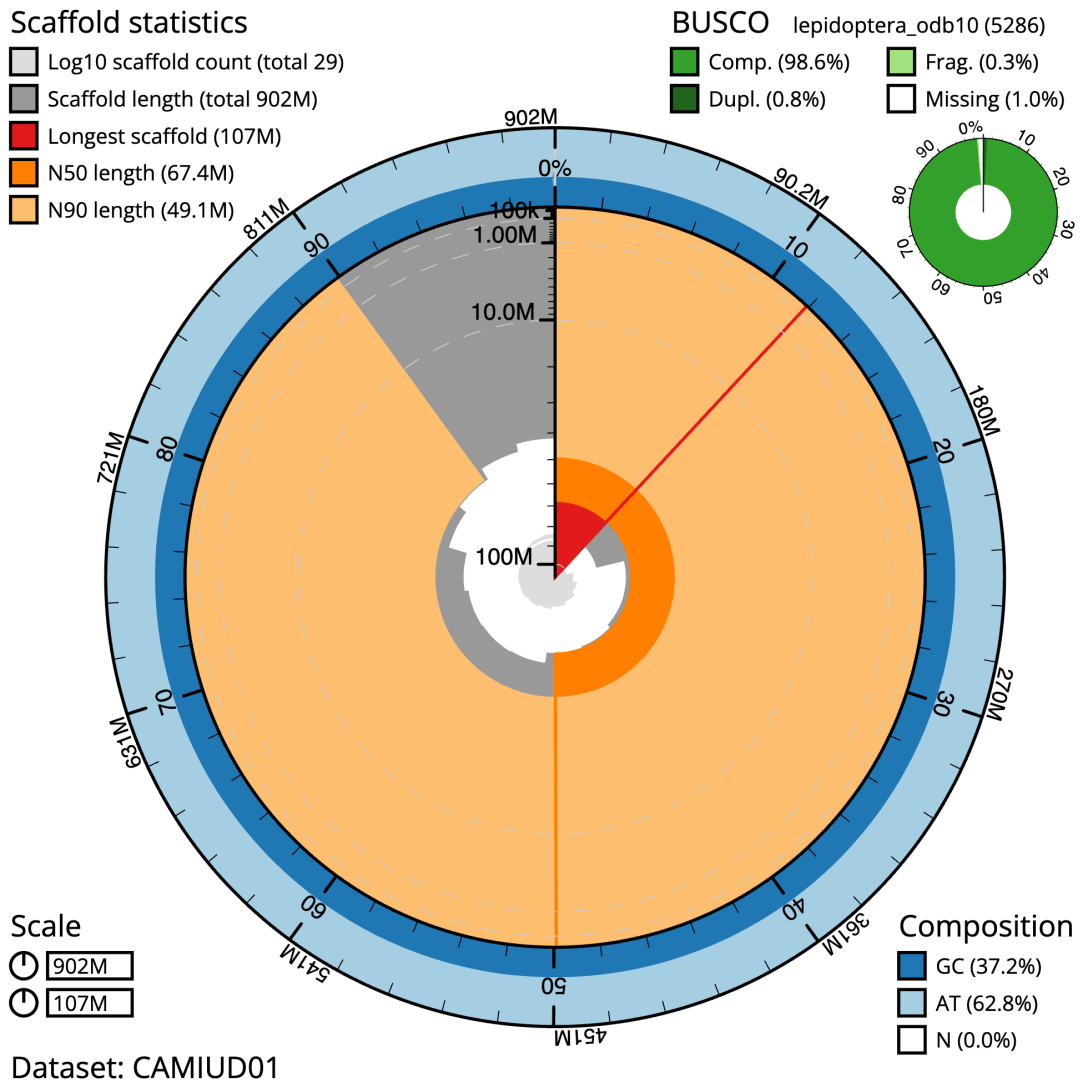
Genome assembly of *Alsophila aescularia*, ilAlsAesc1.1: metrics. The BlobToolKit Snailplot shows N50 metrics and BUSCO gene completeness. The main plot is divided into 1,000 size-ordered bins around the circumference with each bin representing 0.1% of the 901,612,816 bp assembly. The distribution of scaffold lengths is shown in dark grey with the plot radius scaled to the longest scaffold present in the assembly (107,309,733 bp, shown in red). Orange and pale-orange arcs show the N50 and N90 scaffold lengths (67,363,832 and 49,117,080 bp), respectively. The pale grey spiral shows the cumulative scaffold count on a log scale with white scale lines showing successive orders of magnitude. The blue and pale-blue area around the outside of the plot shows the distribution of GC, AT and N percentages in the same bins as the inner plot. A summary of complete, fragmented, duplicated and missing BUSCO genes in the lepidoptera_odb10 set is shown in the top right. An interactive version of this figure is available at https://blobtoolkit.genomehubs.org/view/Alsophila%20aescularia/dataset/CAMIUD01/snail.

**Figure 3.**
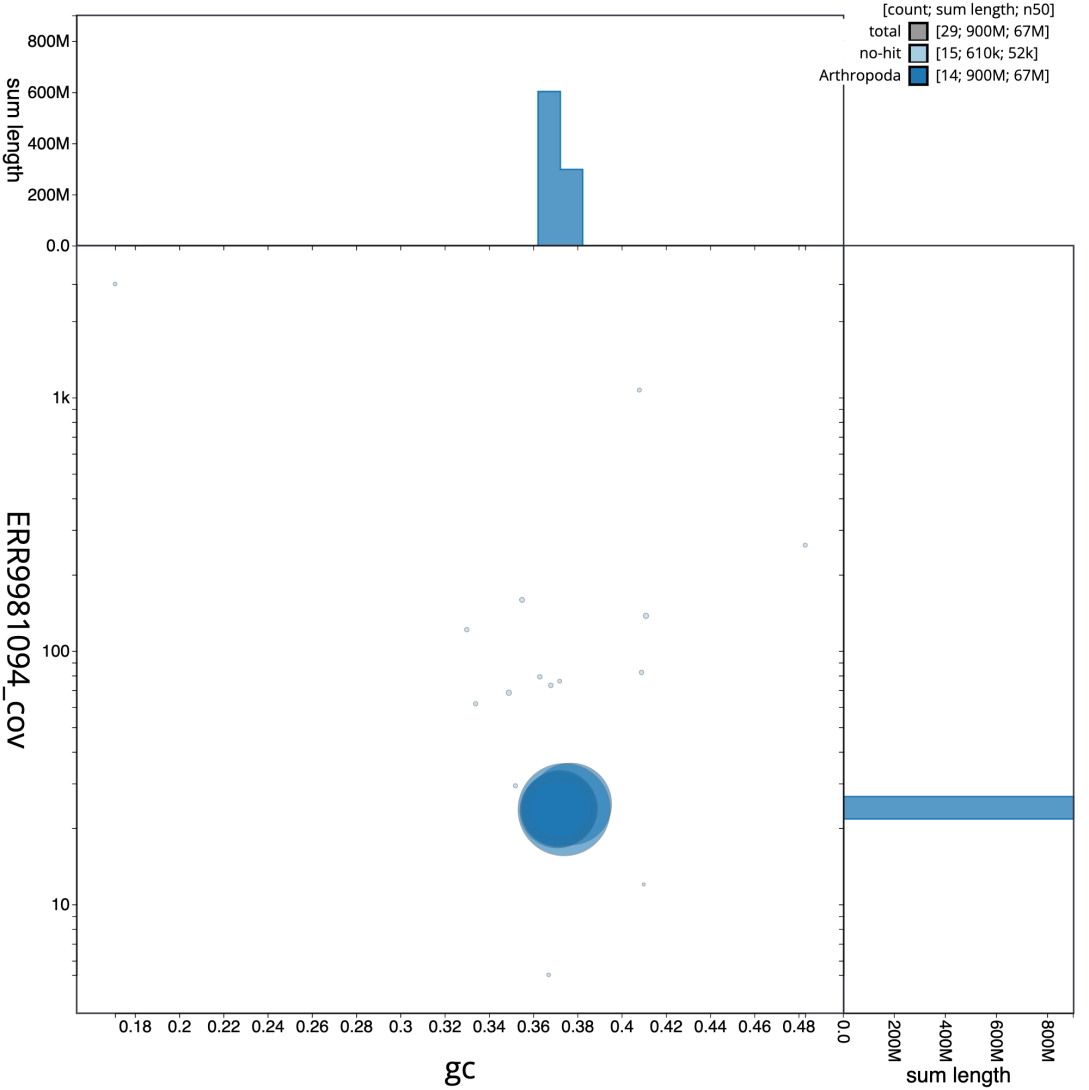
Genome assembly of *Alsophila aescularia*, ilAlsAesc1.1: BlobToolKit GC-coverage plot. Scaffolds are coloured by phylum. Circles are sized in proportion to scaffold length. Histograms show the distribution of scaffold length sum along each axis. An interactive version of this figure is available at https://blobtoolkit.genomehubs.org/view/Alsophila%20aescularia/dataset/CAMIUD01/blob.

**Figure 4.**
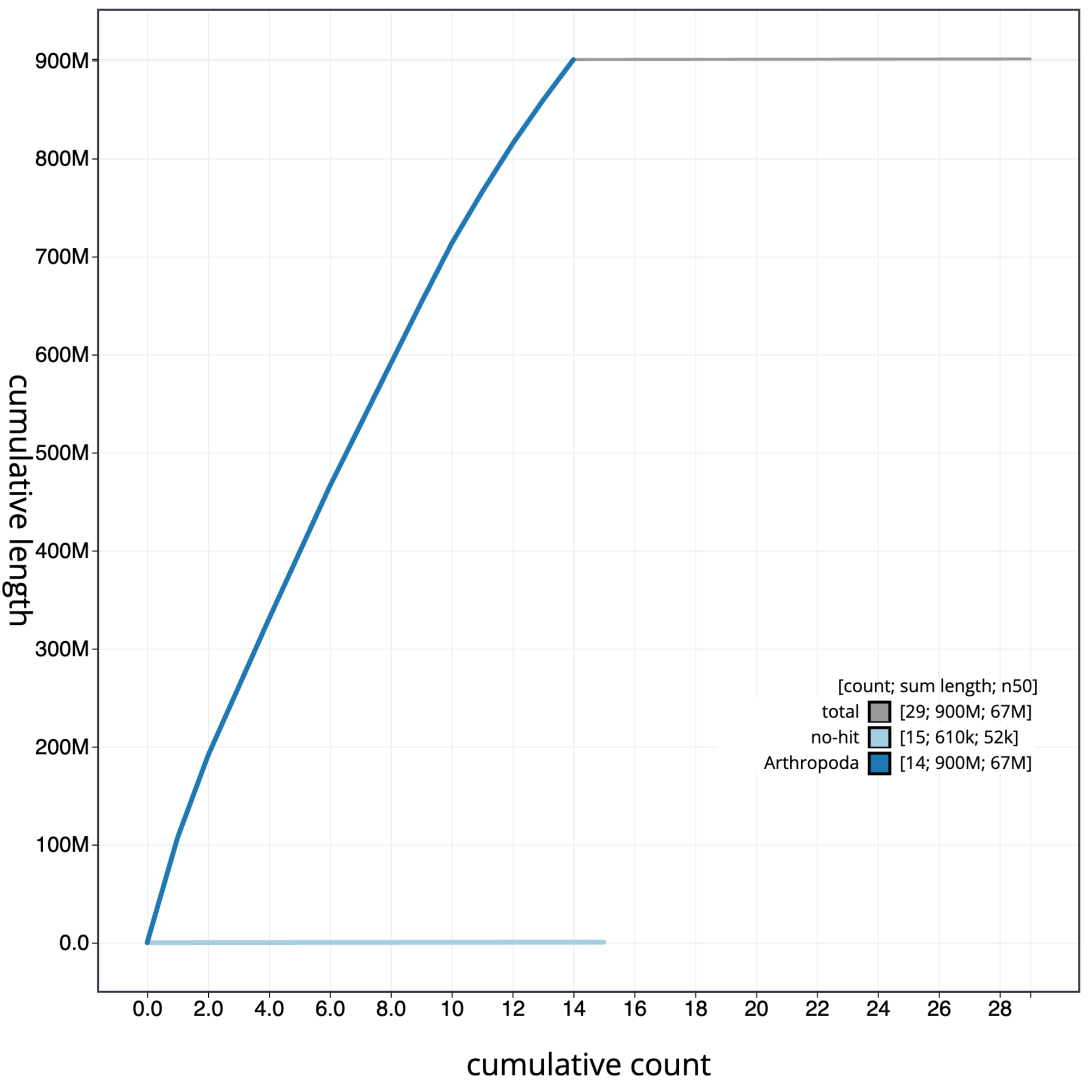
Genome assembly of *Alsophila aescularia*, ilAlsAesc1.1: BlobToolKit cumulative sequence plot. The grey line shows cumulative length for all scaffolds. Coloured lines show cumulative lengths of scaffolds assigned to each phylum using the buscogenes taxrule. An interactive version of this figure is available at https://blobtoolkit.genomehubs.org/view/Alsophila%20aescularia/dataset/CAMIUD01/cumulative.

**Figure 5.**
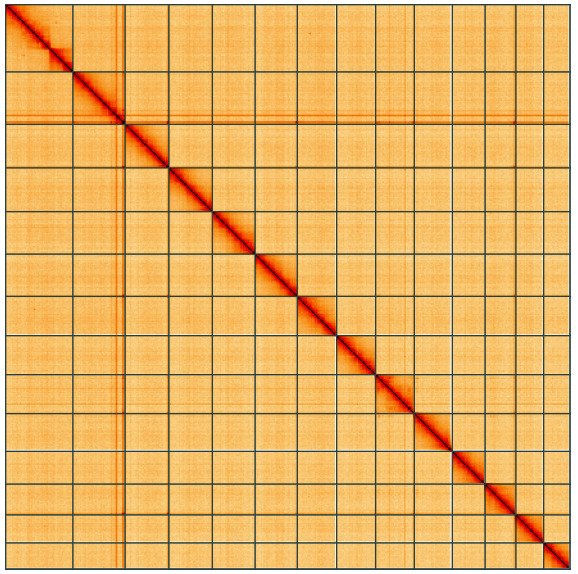
Genome assembly of *Alsophila aescularia*, ilAlsAesc1.1: Hi-C contact map of the ilAlsAesc1.1 assembly, visualised using HiGlass. Chromosomes are shown in order of size from left to right and top to bottom. An interactive version of this figure may be viewed at https://genome-note-higlass.tol.sanger.ac.uk/l/?d=Zzm2cXQWQAyyvZr4TFTIsA.

The final assembly has a total length of 901.6 Mb in 28 sequence scaffolds with a scaffold N50 of 67.4 Mb ( Table 1). The snailplot in Figure 2 provides a summary of the assembly statistics, while the distribution of assembly scaffolds on GC proportion and coverage is shown in Figure 3. The cumulative assembly plot in Figure 4 shows curves for subsets of scaffolds assigned to different phyla. Most (99.93%) of the assembly sequence was assigned to 14 chromosomal-level scaffolds, representing 13 autosomes and the Z sex chromosome. Chromosome-scale scaffolds confirmed by the Hi-C data are named in order of size ( Figure 5; Table 2). While not fully phased, the assembly deposited is of one haplotype. Contigs corresponding to the second haplotype have also been deposited. The mitochondrial genome was also assembled and can be found as a contig within the multifasta file of the genome submission.

**Table 1.** Genome data for *Alsophila aescularia*, ilAlsAesc1.1.

Project accession data		
Assembly identifier	ilAlsAesc1.1	
Species	*Alsophila aescularia*	
Specimen	ilAlsAesc1	
NCBI taxonomy ID	104486	
BioProject	PRJEB54803	
BioSample ID	SAMEA10107017	
Isolate information	ilAlsAesc1, male: thorax (DNA), abdomen (RNA), head (Hi-C)	
Assembly metrics *		*Benchmark*
Consensus quality (QV)	61	*≥ 50*
*k*-mer completeness	100%	*≥ 95%*
BUSCO **	C:98.6%[S:97.8%,D:0.8%],F:0.3%,M:1.0%,n:5,286	*C ≥ 95%*
Percentage of assembly mapped to chromosomes	99.93%	*≥ 95%*
Sex chromosomes	Z chromosome	*localised homologous pairs*
Organelles	Mitochondrial genome assembled	*complete single alleles*
Raw data accessions		
PacificBiosciences SEQUEL II	ERR9981094	
Hi-C Illumina	ERR9988136	
PolyA RNA-Seq Illumina	ERR10378023	
Genome assembly		
Assembly accession	GCA_946251855.1	
*Accession of alternate haplotype*	GCA_946251895.1	
Span (Mb)	901.6	
Number of contigs	140	
Contig N50 length (Mb)	13.3	
Number of scaffolds	28	
Scaffold N50 length (Mb)	67.4	
Longest scaffold (Mb)	107.3	
**Genome annotation**		
Number of protein-coding genes	13,618	
Number of non-coding genes	2,364	
Number of gene transcripts	26,110	

* Assembly metric benchmarks are adapted from column VGP-2020 of “ Table 1: Proposed standards and metrics for defining genome assembly quality” from ( Rhie *et al.*, 2021). ** BUSCO scores based on the lepidoptera_odb10 BUSCO set using v5.3.2. C = complete [S = single copy, D = duplicated], F = fragmented, M = missing, n = number of orthologues in comparison. A full set of BUSCO scores is available at https://blobtoolkit.genomehubs.org/view/Alsophila%20aescularia/dataset/CAMIUD01/busco.

**Table 2.** Chromosomal pseudomolecules in the genome assembly of *Alsophila aescularia*, ilAlsAesc1.

INSDC accession	Chromosome	Length (Mb)	GC%
OX276374.1	1	83.69	37.5
OX276375.1	2	69.7	37.0
OX276376.1	3	69.65	37.0
OX276377.1	4	68.12	37.0
OX276378.1	5	67.36	37.0
OX276379.1	6	62.72	37.5
OX276380.1	7	62.37	37.0
OX276381.1	8	62.06	37.0
OX276382.1	9	60.34	37.0
OX276383.1	10	52.48	37.0
OX276384.1	11	49.12	37.0
OX276385.1	12	44.52	37.5
OX276386.1	13	41.58	37.0
OX276373.1	Z	107.31	37.5
OX276387.1	MT	0.02	17.0

The estimated Quality Value (QV) of the final assembly is 61 with *k*-mer completeness of 100%, and the assembly has a BUSCO v5.3.2 completeness of 98.6% (single = 97.8%, duplicated = 0.8%), using the lepidoptera_odb10 reference set ( *n* = 5,286).

Metadata for specimens, barcode results, spectra estimates, sequencing runs, contaminants and pre-curation assembly statistics are given at https://links.tol.sanger.ac.uk/species/104486.

## Genome annotation report

The *Alsophila aescularia* genome assembly (GCA_946251855.1) was annotated using the Ensembl rapid annotation pipeline ( Table 1; https://rapid.ensembl.org/Alsophila_aescularia_GCA_946251855.1/Info/Index). The resulting annotation includes 26,110 transcribed mRNAs from 13,618 protein-coding and 2,364 non-coding genes.

## Methods

### Sample acquisition and nucleic acid extraction

A male *Alsophila aescularia* (specimen ID Ox001094, ToLID ilAlsAesc1) was collected in a light trap from Wytham Woods, Oxfordshire (biological vice-county Berkshire), UK (latitude 51.77, longitude –1.34) on 2021-03-31. The specimen was collected and identified by Douglas Boyes (University of Oxford) and preserved on dry ice.

The workflow for high molecular weight (HMW) DNA extraction at the Wellcome Sanger Institute (WSI) includes a sequence of core procedures: sample preparation; sample homogenisation, DNA extraction, fragmentation, and clean-up, and the full protocols have been deposited in the protocols.io repository ( Denton *et al.*, 2023b). The sample was prepared for DNA extraction at the WSI Tree of Life laboratory: the ilAlsAesc1 sample was weighed and dissected on dry ice ( Jay *et al.*, 2023), with tissue set aside for Hi-C sequencing. Tissue from the thorax was homogenised using a PowerMasher II tissue disruptor ( Denton *et al.*, 2023a). DNA was extracted at the WSI Scientific Operations core using the Qiagen MagAttract HMW DNA kit, according to the manufacturer’s instructions. HMW DNA was sheared into an average fragment size of 12–20 kb in a Megaruptor 3 system with speed setting 30 ( Todorovic *et al.*, 2023). Sheared DNA was purified by solid-phase reversible immobilisation ( Strickland *et al.*, 2023): In brief, the method employs a 1.8X ratio of AMPure PB beads to sample to eliminate shorter fragments and concentrate the DNA. The concentration of the sheared and purified DNA was assessed using a Nanodrop spectrophotometer and Qubit Fluorometer and Qubit dsDNA High Sensitivity Assay kit. Fragment size distribution was evaluated by running the sample on the FemtoPulse system.

RNA was extracted from abdomen tissue of ilAlsAesc1 in the Tree of Life Laboratory at the WSI using the RNA Extraction: Automated MagMax™ mirVana protocol ( do Amaral *et al.*, 2023). The RNA concentration was assessed using a Nanodrop spectrophotometer and Qubit Fluorometer using the Qubit RNA Broad-Range (BR) Assay kit. Analysis of the integrity of the RNA was done using the Agilent RNA 6000 Pico Kit and Eukaryotic Total RNA assay.

### Sequencing

Pacific Biosciences HiFi circular consensus DNA sequencing libraries were constructed according to the manufacturers’ instructions. Poly(A) RNA-Seq libraries were constructed using the NEB Ultra II RNA Library Prep kit. DNA and RNA sequencing were performed by the Scientific Operations core at the WSI on Pacific Biosciences SEQUEL II (HiFi) and Illumina NovaSeq 6000 (RNA-Seq) instruments. Hi-C data were also generated from head tissue of ilAlsAesc1 using the Arima2 kit and sequenced on the Illumina NovaSeq 6000 instrument.

### Genome assembly, curation and evaluation

Assembly was carried out with Hifiasm ( Cheng *et al.*, 2021) and haplotypic duplication was identified and removed with purge_dups ( Guan *et al.*, 2020). The assembly was then scaffolded with Hi-C data ( Rao *et al.*, 2014) using YaHS ( Zhou *et al.*, 2023). The assembly was checked for contamination and corrected as described previously ( Howe *et al.*, 2021). Manual curation was performed using HiGlass ( Kerpedjiev *et al.*, 2018) and Pretext ( Harry, 2022). The mitochondrial genome was assembled using MitoHiFi ( Uliano-Silva *et al.*, 2023), which runs MitoFinder ( Allio *et al.*, 2020) or MITOS ( Bernt *et al.*, 2013) and uses these annotations to select the final mitochondrial contig and to ensure the general quality of the sequence.

A Hi-C map for the final assembly was produced using bwa-mem2 ( Vasimuddin *et al.*, 2019) in the Cooler file format ( Abdennur & Mirny, 2020). To assess the assembly metrics, the *k*-mer completeness and QV consensus quality values were calculated in Merqury ( Rhie *et al.*, 2020). This work was done using Nextflow ( Di Tommaso *et al.*, 2017) DSL2 pipelines “sanger-tol/readmapping” ( Surana *et al.*, 2023a) and “sanger-tol/genomenote” ( Surana *et al.*, 2023b). The genome was analysed within the BlobToolKit environment ( Challis *et al.*, 2020) and BUSCO scores ( Manni *et al.*, 2021; Simão *et al.*, 2015) were calculated.

Table 3 contains a list of relevant software tool versions and sources.

**Table 3.** Software tools: versions and sources.

Software tool	Version	Source
BlobToolKit	4.1.7	https://github.com/blobtoolkit/blobtoolkit
BUSCO	5.3.2	https://gitlab.com/ezlab/busco
Hifiasm	0.16.1-r375	https://github.com/chhylp123/hifiasm
HiGlass	1.11.6	https://github.com/higlass/higlass
Merqury	MerquryFK	https://github.com/thegenemyers/MERQURY.FK
MitoHiFi	2	https://github.com/marcelauliano/MitoHiFi
PretextView	0.2	https://github.com/wtsi-hpag/PretextView
purge_dups	1.2.3	https://github.com/dfguan/purge_dups
sanger-tol/ genomenote	v1.0	https://github.com/sanger-tol/genomenote
sanger-tol/ readmapping	1.1.0	https://github.com/sanger-tol/readmapping/tree/1.1.0
YaHS	yahs- 1.1.91eebc2	https://github.com/c-zhou/yahs

### Genome annotation

The Ensembl gene annotation system ( Aken *et al.*, 2016) was used to generate annotation for the *Alsophila aescularia* assembly (GCA_946251855.1). Annotation was created primarily through alignment of transcriptomic data to the genome, with gap filling via protein-to-genome alignments of a select set of proteins from UniProt ( UniProt Consortium, 2019).

### Wellcome Sanger Institute – Legal and Governance

The materials that have contributed to this genome note have been supplied by a Darwin Tree of Life Partner. The submission of materials by a Darwin Tree of Life Partner is subject to the **‘Darwin Tree of Life Project Sampling Code of Practice’**, which can be found in full on the Darwin Tree of Life website here. By agreeing with and signing up to the Sampling Code of Practice, the Darwin Tree of Life Partner agrees they will meet the legal and ethical requirements and standards set out within this document in respect of all samples acquired for, and supplied to, the Darwin Tree of Life Project.

Further, the Wellcome Sanger Institute employs a process whereby due diligence is carried out proportionate to the nature of the materials themselves, and the circumstances under which they have been/are to be collected and provided for use. The purpose of this is to address and mitigate any potential legal and/or ethical implications of receipt and use of the materials as part of the research project, and to ensure that in doing so we align with best practice wherever possible. The overarching areas of consideration are:

•   Ethical review of provenance and sourcing of the material

•   Legality of collection, transfer and use (national and international)

Each transfer of samples is further undertaken according to a Research Collaboration Agreement or Material Transfer Agreement entered into by the Darwin Tree of Life Partner, Genome Research Limited (operating as the Wellcome Sanger Institute), and in some circumstances other Darwin Tree of Life collaborators.

## Data Availability

European Nucleotide Archive: *Alsophila aescularia* (March moth). Accession number PRJEB54803; https://identifiers.org/ena.embl/PRJEB54803 ( Wellcome Sanger Institute, 2022). The genome sequence is released openly for reuse. The *Alsophila aescularia* genome sequencing initiative is part of the Darwin Tree of Life (DToL) project. All raw sequence data and the assembly have been deposited in INSDC databases. Raw data and assembly accession identifiers are reported in Table 1.

## Author information

Members of the University of Oxford and Wytham Woods Genome Acquisition Lab are listed here: https://doi.org/10.5281/zenodo.7125292.

Members of the Darwin Tree of Life Barcoding collective are listed here: https://doi.org/10.5281/zenodo.4893703.

Members of the Wellcome Sanger Institute Tree of Life Management, Samples and Laboratory team are listed here: https://doi.org/10.5281/zenodo.10066175.

Members of Wellcome Sanger Institute Scientific Operations: Sequencing Operations are listed here: https://doi.org/10.5281/zenodo.10043364.

Members of the Wellcome Sanger Institute Tree of Life Core Informatics team are listed here: https://doi.org/10.5281/zenodo.10066637.

Members of the Tree of Life Core Informatics collective are listed here: https://doi.org/10.5281/zenodo.5013541.

Members of the Darwin Tree of Life Consortium are listed here: https://doi.org/10.5281/zenodo.4783558.
