# Supplementary material for: The genome sequence of the March moth, Alsophila aescularia (Denis & Schiffermüller)
Source: Wellcome Open Res. 2025 Sep 26;9:50. Originally published 2024 Feb 19. [Version 2] doi: 10.12688/wellcomeopenres.20650.2 (PMC12592854; doi:10.12688/wellcomeopenres.20650.2)
Supplement: Peer review history

# Reviewer report: Reviewer response for version 2

**DOI:** 10.21956/wellcomeopenres.27545.r134781

**Published:** 2025-11-06

**Authors:** Daniel Doucet

**Affiliations:** 1 Natural Resources Canada, Ontario, Canada

The authors describe the complete sequencing and assembly of the genome from the March moth, *Alsophila aescularia. *The moth belongs to one of the most speciose family of the order Lepoidoptera, the geometridae, and as such this genome provides critical insight into the evolution of this important taxa. 

 The rationale for creating the dataset is clearly described. *A. aescularia *is not only present in the UK but also widespread across Europe, thus providing foundational data for future population genomics studies that factor in variables such as climate and geography. The species being polyphagous, host range, host range x climate etc. interactions can also be studied through the lens of population genomics.

 Adequate details on the material and methods of genome sequencing and assembly have been given by the authors. Sequencing methods (PacBio HiFi and Hi-C sequencing), assembly software and workflows are described succinctly, but with enough details such that other researchers with bioinformatics training can replicate the study.

 The datasets provided are standard for Wellcome Open genome report publications, including basic genome assembly statistics (genome length, scaffold N50, BUSCO score, etc.). The mapping of the Merian elements is a nice addition to this type of report, as they allow quick comparison of ancestral linkage groups between lepidopteran sequenced genomes.

 One suggestion I would have for the authors, if they wanted to extend the reach of their report, is to compare the number of *roadkill* ( *rdx*) genes in the genome of *A. aescularia *with that of another geometrid with wingless females, the winter moth ( *Operophtera brumata*). Four *rdx* orthology groups have been identified in insects, and *O. brumea* displays a large and specific  expansion of 25 genes in one particular group, possibly linking *rdx* function with brachyptery. It would be interesting to know if such an expansion is conserved among brachypterous Geometrids.  In *Drosophila melanogaster*, *rdx* genes play a role in organ growth and patterning, particularly in the eye and *rdx* overexpression has been observe to lead to smaller wing size (see citation).

Are sufficient details of methods and materials provided to allow replication by others?

Yes

Is the rationale for creating the dataset(s) clearly described?

Yes

Are the datasets clearly presented in a useable and accessible format?

Yes

Are the protocols appropriate and is the work technically sound?

Yes

Reviewer Expertise:

Insect cell biology

I confirm that I have read this submission and believe that I have an appropriate level of expertise to confirm that it is of an acceptable scientific standard.

## References

[1] : The Genome of Winter Moth (Operophtera brumata ) Provides a Genomic Perspective on Sexual Dimorphism and Phenology. *Genome Biology and Evolution* .2015;7(8) : 10.1093/gbe/evv145 2321-2332 10.1093/gbe/evv145 PMC455886226227816

# Reviewer report: Reviewer response for version 2

**DOI:** 10.21956/wellcomeopenres.27545.r134780

**Published:** 2025-11-06

**Authors:** Sophie Tandonnet

**Affiliations:** 1 Universitat de Barcelona Institut de Recerca de la Biodiversitat (Ringgold ID: 535620), Barcelona, Catalonia, Spain

This note presents a high-quality, chromosome-scale genome assembly of the unusual moth *Alsophila aescularia*, a species notable for its extreme sexual dimorphism and its reduced chromosome number (14 compared to the typical 31 in other lepidopteran species). The mechanisms of sex determination and differentiation, as well as the broader biology of this species, are likely to be particularly intriguing, and this genome will represent a valuable resource for the research community.

 In agreement with the other reviewers, I regret that the heterogametic sex was not sequenced.

 The Z chromosome was identified based on “homologous pairs,” and while it does appear to contain the majority of Merian element MZ, chromosome assignment by homology (the precise method of which is not clearly described here) can be unreliable. I would therefore advise caution and recommend that the authors explicitly state that the sex chromosome assignment remains unverified and may be incorrect.

Are sufficient details of methods and materials provided to allow replication by others?

Yes

Is the rationale for creating the dataset(s) clearly described?

Yes

Are the datasets clearly presented in a useable and accessible format?

Yes

Are the protocols appropriate and is the work technically sound?

Yes

Reviewer Expertise:

Evolutionary Biology, non-model organisms, genomics, sex determination systems

I confirm that I have read this submission and believe that I have an appropriate level of expertise to confirm that it is of an acceptable scientific standard.

# Reviewer report: Reviewer response for version 2

**DOI:** 10.21956/wellcomeopenres.27545.r134416

**Published:** 2025-09-29

**Authors:** Jeffrey Marcus

**Affiliations:** 1 University of Manitoba, Winnipeg, Manitoba, Canada

I thank the authors for responding to the reviews.  They appear to have adequately addressed the critiques of my previous review.

Are sufficient details of methods and materials provided to allow replication by others?

Partly

Is the rationale for creating the dataset(s) clearly described?

Yes

Are the datasets clearly presented in a useable and accessible format?

Yes

Are the protocols appropriate and is the work technically sound?

Yes

Reviewer Expertise:

Evolutionary biology of insects, phylogenomics

I confirm that I have read this submission and believe that I have an appropriate level of expertise to confirm that it is of an acceptable scientific standard.

# Reviewer report: Reviewer response for version 1

**DOI:** 10.21956/wellcomeopenres.22854.r86299

**Published:** 2024-07-05

**Authors:** Jeffrey Marcus

**Affiliations:** 1 University of Manitoba, Winnipeg, Manitoba, Canada

In this manuscript, the authors describe the sequencing and assembly of the *Alsophila aescularia *genome using DNA from a single male specimen collected in the UK.  The primary genome sequence assembly includes proposed chromosomal pseudomolecule sequences for 13 autosomes, the Z sex chromosome, and a complete mitochondrial genome. No females were collected, so the W sex chromosome was not sequenced *.* Overall, this is a reasonably solid contribution to the scientific literature, but due the absence of a W chromosome assembly, the data presented will be limited in its application to “facilitate research into the evolution of sexual dimorphism in wind development”, one of the purported rationales for assembling this genome.

 Some suggestions to the authors:

1. Methods: The date of collection for the sequenced *Alsophila aescularia* specimen is not included in the manuscript. As I have previously noted in a review of a different paper by these same authors [Marcus J. Peer Review Report For: The genome sequence of the bramble shoot moth, Notocelia uddmanniana (Linnaeus, 1758) [version 1; peer review: 1 approved, 1 approved with reservations]. Wellcome Open Res 2021, 6:348 ( https://doi.org/10.21956/wellcomeopenres.19338.r79177)], “it is best practice to include collection dates for all wild-caught insect specimens used for genome sequencing because it is not unusual for cryptic species pairs to be distinguishable by differences in phenology of adult emergence. If it should come to pass that genus [ *Alsophila *] includes cryptic species, knowing the specimen collection date can help future researchers determine to which taxon this sequenced genome should be assigned”. The collection date of the specimen should be added in a revision of this manuscript.
2. And as I have also recommended before, “For future work, I suggest that the researchers preferentially sequence the heterogametic sex when assembling genomes for previously unstudied species (in the case of Lepidoptera, the heterogametic sex is female), so that draft assembles can be prepared for both sex chromosomes.” This is particularly important if authors hope that their work will be used by those who wish to conduct meaningful studies of the genetic basis of sexual dimorphism.
3. I am in agreement with another review of this manuscript by DePrins [De Prins J. Peer Review Report For: The genome sequence of the March moth, Alsophila aescularia (Denis & Schiffermüller) [version 1; peer review: 1 approved with reservations]. Wellcome Open Res 2024, 9:50 ( https://doi.org/10.21956/wellcomeopenres.22854.r78079)] which describes the reconstruction of a genome with 13 autosomes, plus the Z chromosome to be an exceptionally noteworthy finding.  Most Lepidoptera have a haploid chromosome number of 29 (28 autosomes, plus the Z/W sex chromosome), with deeply conserved synteny across lepidopteran taxonomy [Wright, C.J., Stevens, L., Mackintosh, A. *et al.* Comparative genomics reveals the dynamics of chromosome evolution in Lepidoptera. *Nat Ecol Evol* **8**, 777–790 (2024). https://doi.org/10.1038/s41559-024-02329-4].  DePrins has suggested imaging a meiotic plate from this species to confirm the genome structure. That would be helpful, but even a bioinformatics approach to analyzing the existing data set, mapping the sequence synteny between the assembled chromosomes of *Alsophila aescularia* and those of a lepidopteran with a more conventional haploid chromosome number would do much to further our understanding of the chromosomal fusions that have occurred in this instance *. *I encourage the authors to conduct this synteny analysis in a revision of this manuscript.

Are sufficient details of methods and materials provided to allow replication by others?

Partly

Is the rationale for creating the dataset(s) clearly described?

Yes

Are the datasets clearly presented in a useable and accessible format?

Yes

Are the protocols appropriate and is the work technically sound?

Yes

Reviewer Expertise:

Evolutionary biology of insects, phylogenomics, evolution and development of insect wings

I confirm that I have read this submission and believe that I have an appropriate level of expertise to confirm that it is of an acceptable scientific standard, however I have significant reservations, as outlined above.

# Response

**Published:** 2025-09-21

**Authors:** Tree of Life Team Sanger

**Affiliations:** Wellcome Trust Sanger Institute, UK

Thank you for reviewing this data note. Your comments are addressed below.

1. **Methods: The date of collection for the sequenced *Alsophila aescularia* specimen is not included in the manuscript. As I have previously noted in a review of a different paper by these same authors [Marcus J. Peer Review Report For: The genome sequence of the bramble shoot moth, Notocelia uddmanniana (Linnaeus, 1758) [version 1; peer review: 1 approved, 1 approved with reservations]. Wellcome Open Res 2021, 6:348 ( https://doi.org/10.21956/wellcomeopenres.19338.r79177)], “it is best practice to include collection dates for all wild-caught insect specimens used for genome sequencing because it is not unusual for cryptic species pairs to be distinguishable by differences in phenology of adult emergence. If it should come to pass that genus [ *Alsophila *] includes cryptic species, knowing the specimen collection date can help future researchers determine to which taxon this sequenced genome should be assigned”. The collection date of the specimen should be added in a revision of this manuscript.**
*Response: The collection date is given in the Methods section already. Note that the Methods comes after the Genome sequence report and the sample collection information is given in detail under the subheading “Sample acquisition and nucleic acid extraction”.*

 
**2. And as I have also recommended before, “For future work, I suggest that the researchers preferentially sequence the heterogametic sex when assembling genomes for previously unstudied species (in the case of Lepidoptera, the heterogametic sex is female), so that draft assembles can be prepared for both sex chromosomes.” This is particularly important if authors hope that their work will be used by those who wish to conduct meaningful studies of the genetic basis of sexual dimorphism.**

*Response: We agree that it is most useful to sequence the heterogametic sex, and request that collectors provide these as far as possible. * 

**3.I am in agreement with another review of this manuscript by DePrins [De Prins J. Peer Review Report For: The genome sequence of the March moth, Alsophila aescularia (Denis & Schiffermüller) [version 1; peer review: 1 approved with reservations]. Wellcome Open Res 2024, 9:50 ( https://doi.org/10.21956/wellcomeopenres.22854.r78079)] which describes the reconstruction of a genome with 13 autosomes, plus the Z chromosome to be an exceptionally noteworthy finding.  Most Lepidoptera have a haploid chromosome number of 29 (28 autosomes, plus the Z/W sex chromosome), with deeply conserved synteny across lepidopteran taxonomy [Wright, C.J., Stevens, L., Mackintosh, A. *et al.* Comparative genomics reveals the dynamics of chromosome evolution in Lepidoptera. *Nat Ecol Evol* 8, 777–790 (2024). https://doi.org/10.1038/s41559-024-02329-4].  DePrins has suggested imaging a meiotic plate from this species to confirm the genome structure. That would be helpful, but even a bioinformatics approach to analyzing the existing data set, mapping the sequence synteny between the assembled chromosomes of *Alsophila aescularia* and those of a lepidopteran with a more conventional haploid chromosome number would do much to further our understanding of the chromosomal fusions that have occurred **in this instance *. *I encourage the authors to conduct this synteny analysis in a revision of this manuscript.**

*Response: We have added a Merian plot (Figure 6) to show how ancestral single-copy orthologues map to the chromosomes here, illustrating the chromosome fusions that have taken place in this lineage.*

# Reviewer report: Reviewer response for version 1

**DOI:** 10.21956/wellcomeopenres.22854.r78079

**Published:** 2024-04-18

**Authors:** urate De Prins

**Affiliations:** 1 Royal Belgian Institute of Natural Sciences, Brussels, Belgium

The correct identification of the specimen that is sequenced with full genome methodology is the first, but essential step of any further study. We need correct evidence-based data, and not the long-read sequences that are obtained of doubtful or even misidentified species. Lepidoptera are identified by the combination of multi-evidence characters that are obtained from external morphology, internal micromorphology, bionomics, and DNA. Some species of Lepidoptera can be identified from external morphology only, but most of them are not. Therefore, it is a very desirable methodological approach in the Tree of Life initiative to include the full possible evidence of correct species identification.

 In this particular case, the species *Alsophila aescularia* is correctly identified by external morphology only. The specimen presented in Figure1 certainly belongs to *Alsophila aescularia. *This species is particular by its extreme sexual dimorphism. Females are wingless. This phenomenon no doubt has an expression in genomics.

 The other super rare fact is that the chromosome number of this species is n=14!  This is very rare in moths (Heterocera) and there is no evidence presented in the paper except the part of the sentence "the assembly sequence was assigned to 14 chromosomal-level scaffolds, representing 13 autosomes and the Z sex chromosome".

 Suggestions:

- Maybe the authors could present a meiotic plate or other visual evidence of such a rare case of chromosome arrangement in Lepidoptera. Is this related to extreme sexual dimorphism or do other mainly genetic factors play a role?   

Questions:

- Is there any genetic factor found in full genome sequence data for extreme sexual dimorphism in *Alsophila aescularia?* 
- Is the extreme fusion of chromosome set in *Alsophila aescularia *which is more than 2 times less than what is a usual case in moths, could be explained somehow by genetic data?

It is likely that these two important evolutionary questions can be answered in further studies.

Are sufficient details of methods and materials provided to allow replication by others?

Yes

Is the rationale for creating the dataset(s) clearly described?

Yes

Are the datasets clearly presented in a useable and accessible format?

Yes

Are the protocols appropriate and is the work technically sound?

Yes

Reviewer Expertise:

Karyology of primitive moths, taxonomy, nomenclature, diversity of tropical taxa of primitive moths, moth-plant relationships, bioinformatics

I confirm that I have read this submission and believe that I have an appropriate level of expertise to confirm that it is of an acceptable scientific standard, however I have significant reservations, as outlined above.

# Response

**Published:** 2025-09-21

**Authors:** Tree of Life Team Sanger

**Affiliations:** Wellcome Trust Sanger Institute, UK

Thank you for reviewing this data note. Your comments are addressed below.  

**Suggestions:**

- Maybe the authors could present a meiotic plate or other visual evidence of such a rare case of chromosome arrangement in Lepidoptera. Is this related to extreme sexual dimorphism or do other mainly genetic factors play a role?   

***Response**: We have added a Merian plot (Figure 6) to show how ancestral single-copy orthologues map to the chromosomes here, illustrating the chromosome fusions that have taken place in this lineage.*

**Questions:**

- Is there any genetic factor found in full genome sequence data for extreme sexual dimorphism in *Alsophila aescularia?* 
- Is the extreme fusion of chromosome set in *Alsophila aescularia *which is more than 2 times less than what is a usual case in moths, could be explained somehow by genetic data?

It is likely that these two important evolutionary questions can be answered in further studies.

***Response**: We agree, and hope that the dataset provided here will be a useful resource for these studies.*
